# Gallstones Successfully Treated with Olive Oil

**Published:** 1883-05-20

**Authors:** F. Loeber


### GALLSTONES SUCCESSFULLY TREATED WITH OLIVE OIL.

Before relating these cases I deem it my duty to say that the treatment about to be mentioned is not original with myself. I got it from a medical journal, published, I believe, in Wisconsin. It is now some years past.

If I remember correctly, the author of the article containing the treatment, excused himself for obtruding one more among the vast number of remedies for gallstone, with the plea that he had suffered from the affliction for several years, and had tried everything recommended by text-books and Journals without avail, and at last had been induced by a neighbor, a farmer, to try sweet oil. He did so, cured himself, and has since cured several other persons of the same painful trouble.

Fortunately for myself, I have never been a sufferer from gallstones, but I take the liberty of once more obtruding this homely, but effective remedy before the medical public.

The treatment as recommended, is to give the patient, after the bowels have been evacuated by a light laxative or water-enema, a large dose of oleum olivæ, ℥ x—xij.

After taking, the patient must remain perfectly quiet in bed, lying on the side with the pelvis slightly elevated for, say, three hours, or until the oil begins to act.

In this way, I have treated five cases—all with the happiest results.

CASE.—J. K., 46 years old, British seaman, entered the Touro Infirmary, suffering from intense icterus, but no fever. Considerable tenderness in right hypochondrium; liver somewhat enlarged. Said that he left the hospital in Liverpool just before shipping for New Orleans, where he had gone with a similar attack of jaundice, which came on after a severe pain in his side and belly.

*Diagnosis*—Gallstone. Patient placed on light diet. Bowels freely evacuated by castor-oil and turpentine. After that, ordered Carlsbad water treatment. All went nicely. The stools began to color, and the icterus to disappear. A week subsequently, at my evening visit, I found the man suffering from violent colic. His abdomen was swollen, and he vomited incessantly. Pulse quick and small; body bathed in perspiration. Ordered warm flax-seed poultice to patient's abdomen. Gave, hypodermically—

Morph. sulph.................................gr. $\frac{1}{3}$,

Atropiæ sulph.................................gr. 1–48.

Repeated the morphine in half an hour but without relief. Then resorted to chloroform inhalations for some two hours, till the pain subsided, and sleep came on.

Next morning, much prostration, intense icterus, white stools. Repeated the castor-oil and turpentine, and instead of Carlsbad water, ordered Durand's treatment—three parts of sulph. ether to two parts ol. terebinth—a teaspoonful every morning.

Things again went on nicely. The stools began to look natural; the skin to lose its yellow hue. In ten days, however, he had another attack, quite as violent as the first, and the same means had to be resorted to, to relieve pain.

That evening, in looking over my journals, I came across the "sweet-oil treatment," and at once ordered the nurse to give the patient a full water injection before my morning visit.

At 9 o'clock a.m., gave him ol. olivæ, ℥ xii, and saw that the position recommended was observed.

In two hours and a half his bowels began to move, and to my astonishment, such a number of stones was passed as to fill two ordinary strawberry baskets—nearly two pints—some as large as walnuts.

I ordered the nurse to clean and put them in a bottle, to be shown at our regular weekly medical meeting. But unfortunately, in his zeal to preserve them, he covered them with alcohol. In consequence they were almost totally dissolved

After a lapse of a week, detecting slight tenderness and a little tumor in the right hypochondriac region, I repeated the oil, with the same result, only not so many stones were passed.

Three weeks later the patient left the hospital apparently well, but took care to provide himself with a bottle of sweet-oil.

The four other cases were three females and one male—one being a lady sixty-five years of age. Nothing besides the olive oil was used on these patients. All of them discharged large quantities of stones, having suffered previously for many years. Since that time they have remained well. All four cases reside in the city, and I am able to keep them under observation.—F. LOEBER, *in N. O. Med. Journal.*

